# A route pruning algorithm for an automated geographic location graph construction

**DOI:** 10.1038/s41598-021-90943-8

**Published:** 2021-06-02

**Authors:** Christoph Schweimer, Bernhard C. Geiger, Meizhu Wang, Sergiy Gogolenko, Imran Mahmood, Alireza Jahani, Diana Suleimenova, Derek Groen

**Affiliations:** 1 Know-Center GmbH, Graz, Austria; 2 High Performance Computing Center Stuttgart, Stuttgart, Germany; 3 Department of Computer Science, Brunel University London, London, UK; 4 Centre for Computational Science, University College London, London, UK

**Keywords:** Computer science, Information technology

## Abstract

Automated construction of location graphs is instrumental but challenging, particularly in logistics optimisation problems and agent-based movement simulations. Hence, we propose an algorithm for automated construction of location graphs, in which vertices correspond to geographic locations of interest and edges to direct travelling routes between them. Our approach involves two steps. In the first step, we use a routing service to compute distances between all pairs of *L* locations, resulting in a complete graph. In the second step, we prune this graph by removing edges corresponding to indirect routes, identified using the triangle inequality. The computational complexity of this second step is $\mathscr{O}(L^3)$, which enables the computation of location graphs for all towns and cities on the road network of an entire continent. To illustrate the utility of our algorithm in an application, we constructed location graphs for four regions of different size and road infrastructures and compared them to manually created ground truths. Our algorithm simultaneously achieved precision and recall values around 0.9 for a wide range of the single hyperparameter, suggesting that it is a valid approach to create large location graphs for which a manual creation is infeasible.

## Introduction

Geographic information systems (GIS) and web mapping have evolved over the past three decades as technological advances enable the developments in geospatial data and mapping usage^1^. Consequently, web mapping services, such as Google Maps, Bing Maps and OpenStreetMap (OSM), have emerged with various functionalities to inspect, visualise, analyse and model geospatial information. These commonly known services are available and accessible by everyone through interactive and visual interfaces, as well as provide an interface to download geographic information, including lists of cities or other points-of-interest within a geographic region, and routes between pairs of locations (if the service also provides routing capabilities).

However, these interfaces have functional limitations, which do not allow the automatic generation of highly customised geographic information. In this work, we consider a relevant subclass of such customised geographic information: location graphs, in which locations of interest are connected by edges if there exists a direct route between them. Such location graphs are a prerequisite for many real-life problems, for example, route optimisation, load optimisation in electrical and transportation networks, and many others. Location graphs are also needed for agent-based modelling applications such as transportation of goods^2^, evacuation models^3,4^, traffic simulations^5^, disease transmission^6^, movement of people^7^, and migration simulation^8^.

Previously, these location graphs were created manually. For example, for the migration study of Suleimenova et al.^8^, the length of a link (in km) was estimated using the OSM route planner for cars. In cases where obvious shorter routes are visible, the mapping marker was dragged to force the routing machine to calculate these shorter routes. Yet, such manual creation of location graphs is a time-consuming and error-prone procedure, and can only be accomplished for small sets of locations.

We propose an automated approach for the construction of location graphs for given lists of locations. This approach relies on a two-step procedure. In the first step, we utilise interfaces provided by mapping services, such as the Open Source Routing Machine (OSRM) from OSM, to compute route distances between all pairs of locations, essentially corresponding to a fully connected location graph. In the second step, edges are pruned from this fully connected location graph that correspond to *indirect* routes, i.e., to routes between two locations that pass through a third location in the location graph. Since the first step yields only a matrix of distances but no further information about the routes, finding these indirect routes is nontrivial. We approach this problem by making use of the triangle inequality: if the route between two locations has a distance similar to the sum of distances of routes between these locations and a common third location, then it is probable that the considered route is indirect. While the first step of our procedure relies on existing algorithms for finding shortest paths in graphs, the second step presents our first contribution in the area of edge pruning algorithms (see “Related work” section).

As our second contribution, we add a real-valued parameter $\beta$ to our pruning algorithm that extends the flexibility of our approach and allows to control the quality of pruning. Specifically, when deciding whether a route shall be pruned, we compare the route distance between two locations to the sum of distances between these locations and a third one, multiplied by $\beta$. Thus, if $\beta >1$, the resulting pruned graph may not be completely pruned, but may rather be redundant by retaining edges corresponding to sub-optimal routes (i.e., with longer distances). If instead $0<\beta <1$, then the resulting graph is lossy in the sense that not all shortest paths are retained. Thus, the parameter $\beta$ allows trading between the quality (in terms of redundancy and path quality) and complexity (in terms of edge set size) of the simplified graph. As a consequence, our approach complements and extends the work of Zhou et al.^9,10^, who approached graph simplification by pruning a given number of edges such that path quality is maximised while we control the quality of the lossy pruning by the relaxation parameter $0<\beta \le 1$.

Our third contribution is to validate the applicability of our approach and investigate its limitations by applying it to four different scenarios. We constructed location graphs for two small regions in Europe and for two large regions in Africa, respectively, and compared the results to manually created ground truths. In three of the four regions, we achieved an F1-score (see “Results” section for a definition) exceeding 0.9 for the same value of the single parameter of our method. We furthermore showed that our approach scales well to larger location sets, thus enabling the creation of location graphs with tens of thousands of locations. The implementation of our method is available at https://github.com/djgroen/ExtractMap.

## Related work

The shortest path algorithms for **route planning** can be categorised into static, dynamic, time-dependent, stochastic, parametric, alternative and weighted region shortest-path algorithms^11,12^. These algorithms establish the algorithmic basis for state-of-the-art route planning engines such as Google Maps, Bing Maps, or OSRM. The *static* category includes single-source and all-pairs shortest-path algorithms that differ in terms of a given edge to other edges or all-pairs to other edges in the graph. One of the most known shortest-path algorithms was proposed by Dijkstra^13^. It finds a shortest path between two vertices in a graph. Dijkstra’s algorithm has numerous variations that are commonly applied to speed-up computing and tackle diverse problems of general and complex graphs^11,14^. *Dynamic* algorithms consider insertion and deletion of edges, as well as a computation of the distances between single-source or all-pairs edges in the graph. *Other* categories refer to changes over time, uncertainty in edges, specific parameter values, avoiding given edges and weighted subdivision edges. In this work, we focus our interest on the category of batched shortest path algorithms which are commonly used for computing *distance matrices* in route planning engines^12^.

State-of-the-art *route planning engines* implement an API for finding travel distances and journey duration of fastest routes between all pairs of supplied origins using a given mode of travel. Examples of these include Distance Matrix Service of Google Maps, Distance Matrix API of Bing Maps, and Table Service of OSRM. Online routing services impose different constraints on the size and quantity of such API queries. In particular, Bing API allows up to 2500 origins-destinations pairs, while Google API establishes pricing per origin-destination pair in the Distance Matrix queries. Moreover, online services usually have a limited uncustomizable set of travel modes, which prevents tailoring models for speed of traveller movement on different terrains and road types. Being a free open-source off-line tool, OSRM relaxes these limitations^15^.

*OSRM* implements multilevel Dijkstra’s (MLD) and contraction hierarchies (CH) algorithms for routing^15^. Both methods consist of preprocessing and query phases. The preprocessing phase attempts to annotate and simplify the complicated route network in order to drastically reduce duration of further shortest-path and batched shortest-path queries. MLD belongs to the family of separator-based shortest-path techniques^11,12^. Conceptually, it differs from the celebrated customizable route planning (CRP) algorithm^16,17^ only by the hierarchical partitioning approach used in the preprocessing phase: canonical CRP applies patented graph partitioning with natural cuts (PUNCH) approach, while MLD opts for inertial flow approach^18^. Contraction hierarchies is a classic hierarchical shortest-path algorithm^11,12^, widely discussed in the literature^19,20^.

**Network simplification by edge pruning** emerged in various contexts and has been addressed under different names by a number of authors^9,10,21–25^. Specifically, the authors propose and study a generic path-oriented framework for graph simplification^9,10,25^. This framework targets to simplify a graph by reducing the number of edges while preserving the maximum path quality metric for any pair of vertices in the graph. It covers a broad class of optimisation problems for probabilistic graphs, flow graphs, and distance graphs. Distance graph pruning, as it is investigated in this work, can be viewed as a special case of the path-oriented graph simplification where the inverse of the path length serves as a path quality metric. Toivonen et al.^25^ introduce four generic strategies for lossless path-oriented graph simplification, where the term *lossless* in the context of distance graphs implies that all fastest routes between pairs of locations are preserved in the pruned graph. Later this approach was extended to a *lossy* graph pruning with a given number of edges to remove^9,10^.

Our pruning approach based on the triangle inequality closely relates to the Static-Triangle strategy from Toivonen et al.^25^ which has a time complexity of $\mathscr {O}(L\cdot R)$, where *L* and *R* are the number of locations and routes in the original graph, respectively. For general (potentially sparse) graphs, this strategy is sub-optimal in the sense that the obtained graph may contain redundant routes, and the authors thus also propose an alternative, optimal strategy (called Iterative-Global) with a higher time complexity of $\mathscr {O}(R(R+L)\log L)$. However, for a complete location graph in which route distances satisfy the triangle inequality and ignoring the effect of ties, the Static-Triangle strategy and our own approach can be shown to be optimal in the sense of eliminating all redundant routes. In this case, since $R=L^2$, the time complexity of our approach is $\mathscr {O}(L^3)$, which compares favourably with the time complexity of $\mathscr {O}(L^4\log L)$ of the optimal Iterative-Global strategy^25^. Since the first step of our two-step approach results with a complete location graph where the route distances satisfy the triangle inequality, we can reap the benefits of this reduced time complexity without loss of optimality.

## Methods

We are given a set of *L* locations $\mathscr {L}=\{l_1,\dots ,l_L\}$ in a geographical region. We are interested in a weighted graph $\mathscr {G}=(\mathscr {L},E,D)$ with vertices $\mathscr {L}$, edges *E* corresponding to direct routes between locations, and edge weights *D* corresponding to route distances that keeps only fastest (or shortest) paths between all pairs of vertices from $\mathscr {L}$.

The problem of finding an optimal location graph can be formalised as follows. We assume a weighted, potentially directed route graph $G=(\mathscr {L}_G, E_G)$ with $L_G$ vertices is given. Each edge $e := (u,v) \in E_G$ corresponds to a route connecting two locations *u* and *v* from $\mathscr {L}_G$ and has a positive-valued weight $d_G(e)\in \mathbb {R}^+$ that corresponds to the route distance between *u* and *v*. A path *P* is a sequence of edges, e.g. $P=((u_1,u_2), (u_2,u_3), \ldots , (u_{k-1}, u_k))=:[u_1 - u_2 - \cdots - u_k]$. We denote by $u_1\overset{G}{\leadsto } u_k$ the set of all feasible paths between $u_1$ and $u_k$ in *G*. The length of the *shortest path* between *u* and *v* is thus defined as

1 $$\begin{aligned} Q(u,v; G) = \min _{P \in u \overset{G}{\leadsto } v}\left( \sum _{e \in P} d_G(e)\right) . \end{aligned}$$

For the given subset of locations $\mathscr {L}\subseteq \mathscr {L}_G$, our goal is to find a weighted graph $\mathscr {G}=(\mathscr {L},E)$ with a minimum number of edges such that $Q(u,v; \mathscr {G}) = Q(u,v; G)$ for all $\{u,v\} \subseteq \mathscr {L}$. For the sake of brevity, we limit further discussion to the undirected graphs. Nevertheless, our approach straightforwardly extends to the directed graphs.

To create the graph $\mathscr {G}$, we propose a two-step procedure. In the first step, we use a routing service to find route distances between all pairs of locations. Assuming that the distances are symmetric, we terminate with an undirected fully connected graph $\mathscr {G}^*=(\mathscr {L},[\mathscr {L}]^2,D^*)$, where $[\mathscr {L}]^2$ is the set of two-element subsets of $\mathscr {L}$ and where $D^*=[d_{i,j}^*]$ is the matrix of distances between locations with $d_{i,j}^*=d_{\mathscr {G}^*}(\{l_i,l_j\})$. Many of the distances computed by the route planner will correspond to indirect routes, as a route between two locations in $\mathscr {L}$ may pass through another location in $\mathscr {L}$. Therefore, in a second step, we use the distance matrix $D^*$ to identify edges in $\mathscr {G}^*$ that correspond to redundant routes, and remove them to obtain $\mathscr {G}$. In this section, we will give an overview of this two-step procedure.

### Step 1: Creating a fully connected graph via route planning

For route planning, we rely on map data from OSM, together with the C++ routing machine from the OSRM Project (http://project-osrm.org), i.e., we work with locally downloaded map data and a C++ wrapper for OSRM, allowing requests for large sets of locations $\mathscr {L}$. However, any other routing service can be used, including online services for smaller sets of locations. In our experiments, we obtained pairwise distances between up to $L=18000$ locations. The result is a distance matrix $D^*=[d_{i,j}^*]$, with $d^*_{i,j}$ being the distance between locations $l_i$ and $l_j$. If there is no route between $l_i$ and $l_j$, then the respective distance is set to $\infty$. Throughout this work and in pruning algorithm implementation, we assume that the matrix $D^*$ is symmetric and has an all-zero main diagonal, i.e., ${L(L-1)}/{2}$ degrees of freedom.

### Step 2: Algorithm for pruning redundant routes

Of the ${L(L-1)}/{2}$ route distances obtained in the previous step, a significant portion will represent indirect routes. For example, suppose that locations $l_1$, $l_2$, and $l_3$ lie on the same road in a geographical region, with location $l_2$ lying between the other two. The road network has an edge from $l_1$ to $l_2$ and an edge from $l_2$ to $l_3$, but no direct edge from $l_1$ to $l_3$. Thus, for the construction of the weight matrix $D=[d_{i,j}]$ in our desired graph $\mathscr {G}$, we need to set $d_{1,3}=d_{3,1}=\infty$ and ensure that the edge $\{l_1,l_3\}\not \in E$.

In order to detect indirect routes, we make use of the following reasoning. If $l_2$ lies on the same road and between $l_1$ and $l_3$, then one may expect that $d^*_{1,2}+d^*_{2,3}\approx d^*_{1,3}$. In fact, in most cases we will have $d^*_{1,2}+d^*_{2,3} > d^*_{1,3}$, because $l_2$ may not lie *directly* on the route between $l_1$ and $l_3$. At the same time, if $l_2$ lies on the same road and between $l_1$ and $l_3$, then $d^*_{1,3}$ will be the longest of the three routes, i.e., $d^*_{1,3}\ge \max \{d^*_{1,2},d^*_{2,3}\}$. Thus, if in a triangle of locations $l_i$, $l_j$, and $l_k$ with distances $d^*_{i,j}$, $d^*_{i,k}$, and $d^*_{j,k}$, the largest distance is larger than the sum of the two smaller distances, then it is very likely that the largest distance corresponds to an indirect route, which subsequently is removed from $\mathscr {G}^*$ to arrive at $\mathscr {G}$.

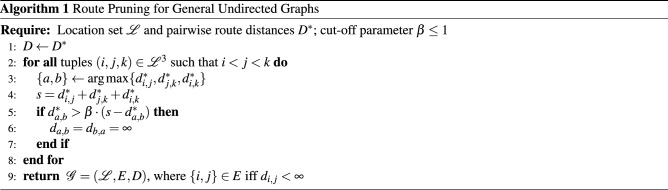



The pseudocode in Algorithm 1 summarises these ideas. Note that, by the restriction that $i<j<k$ in line 2, it only operates on the upper triangle of $D^*$, since we assume that the matrix $D^*$ is symmetric. Since the algorithm iterates over all $L(L-1)(L-2)/6$ possible triples of locations, the computational complexity is $\mathscr {O}(L^3)$.

**Figure 1:**
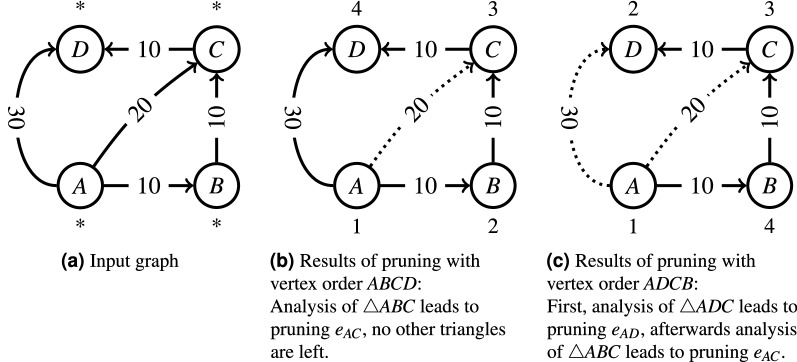
Impact of triplets order on pruning results.

It is important to highlight that Algorithm 1 executes route pruning on a copy of the fully connected graph (see line 1) while checking the pruning condition on the input graph $\mathscr {G}^*$ (see line 5). Otherwise, the triplets order may impact the results of pruning and lead to incorrect conclusions. In particular, Fig. 1 illustrates an example when the natural lexicographic order of triangle traversal leads to incorrect pruning (Fig. 1b), whereas a slightly modified order produces the right answer (Fig. 1c).

As can be seen in line 5 of Algorithm 1, we added a parameter $\beta$ in order to relax the condition posed by the triangle inequality. A value $\beta <1$ allows removing the longest side of a triangular route even if it is slightly shorter than the sum of the two remaining routes. This makes sense if three locations lie along a road, but getting to these locations requires a short detour (e.g. getting off the highway and to the city centre before getting back on the highway). The larger $\beta$, the more conservative is our pruning algorithm. Rather than such a multiplicative relaxation, allowing the largest distance to exceed the sum of the other two distances by some percentage, an additive relaxation is possible as well, or a combination thereof (e.g. by replacing the condition in line 5 by $s-d_{a,b}^*>\min \{d_{a,b}^*/\beta , d_{a,b}^*+\delta \}$, where $\delta$ is a tunable parameter corresponding to an absolute distance).

The idea of triangular pruning extends naturally to sparse or directed input graphs $\mathscr {G}^*=(\mathscr {L},E^*,D^*)$. If the graph is directed, then $E^*$ is a subset of $\mathscr {L}^2$ and $D^*$ need not be symmetric anymore. Such a situation can occur in cases in which distances between locations depend on the direction between them, e.g. caused by one-way streets. If the graph is sparse, then $E^*$ is a proper subset of $\mathscr {L}^2$ (in the directed case) or $[\mathscr {L}]^2$ (in the undirected case). This can be caused by prior information on the road network, for example, or by adjustments made in Step 1 of our approach.

We close this section by showing that Algorithm 1 terminates with a completely pruned graph also in settings different from the one considered here. For general graphs $\mathscr {G}^*$, an edge $\{l_1,l_k\}$ is redundant if and only if there is a path $P=[l_1 - l_2 - \cdots - l_k]$ that is shorter than $d^*_{1,k}$. This consideration is the motivation behind the “Global” strategies of Toivonen et al.^25^ Now suppose that the graph $\mathscr {G}^*$ is complete and satisfies the triangle inequality. In other words, if $P=[l_1 - l_2 - \cdots - l_k]$ is a path in this graph, then for every vertex $l_j$, $j\in \{2,3,\dots ,k-1\}$, we have that the length of *P* in $\mathscr {G}^*$ is at least $d^*_{1,j}+d^*_{j,k}$ (such as in the graph that we obtain in step 1). Then, it is apparent that the edge $\{l_1,l_k\}$ is redundant if and only if there is a location $l_j$ such that $d^*_{1,j}+d^*_{j,k}<d^*_{1,k}$. This shows that for these types of graphs the “Triangle” strategies of Toivonen et al.^25^ and our Algorithm 1 are optimal.

## Results

To validate our route pruning approach, understand its limitations and its dependence on the parameter $\beta$, we tested it in four geographical regions, namely the federal state of Styria in Austria, a region at the German-Austrian border, the Central African Republic and South Sudan countries.

For Step 1 of our approach we relied on OSM map data downloaded from https://download.geofabrik.de and applied an offline version of OSRM to compute route distances (shortest driving time) between several location types, e.g. established cities, small towns and (temporary) refugee camps, in the four considered geographical regions. We subsequently applied Algorithm 1 for Step 2 to obtain the pruned location graph $\mathscr {G}$.

The accuracy of Algorithm 1 w.r.t. a manually created ground truth of direct driving routes is measured in terms of Precision, Recall and F1-score. To calculate these three performance indicators, the number of True Positives (TP), False Positives (FP) and False Negatives (FN) is needed. In our study, a TP is a route that is part of the ground truth and that is also detected by the pruning algorithm, a FP is a route that is not part of the ground truth, but is labelled as a direct route by the pruning algorithm, and a FN is a route that is part of the ground truth, but pruned from the fully connected graph by the algorithm. From these, Precision, Recall, and F1-score are calculated as follows:

$${\text{Precision}} = \frac{{{\text{TP}}}}{{{\text{TP}} + {\text{FP}}}}\qquad {\text{Recall}} = \frac{{{\text{TP}}}}{{{\text{TP}} + {\text{FN}}}}\qquad {\text{F1 - score}} = 2 \cdot \frac{{{\text{Precision}} - {\text{Recall}}}}{{{\text{Precision}} + {\text{Recall}}}}$$

**Figure 2:**
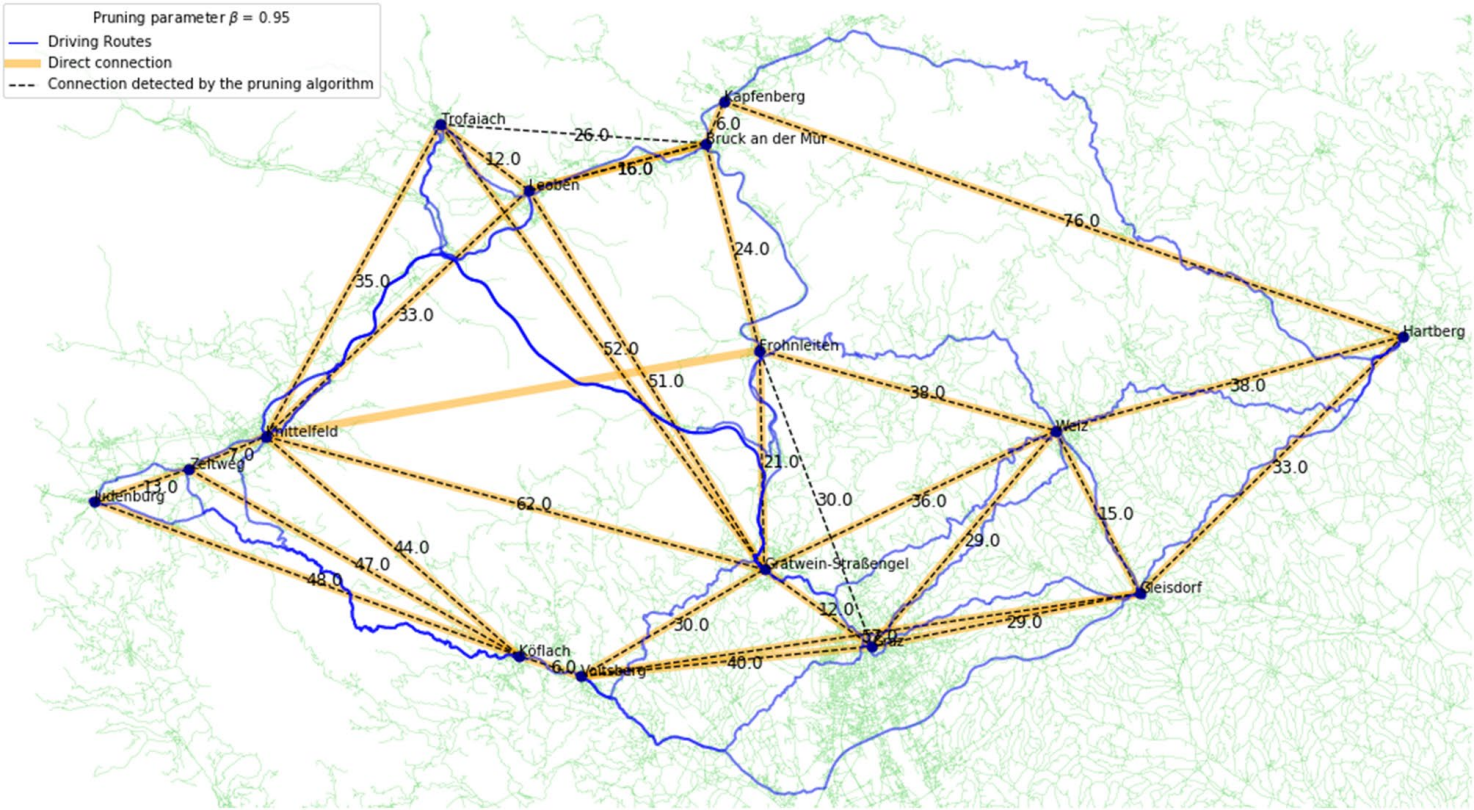
Connections of 15 locations in the federal state of Styria, Austria with $\beta =0.95$.

**Figure 3:**
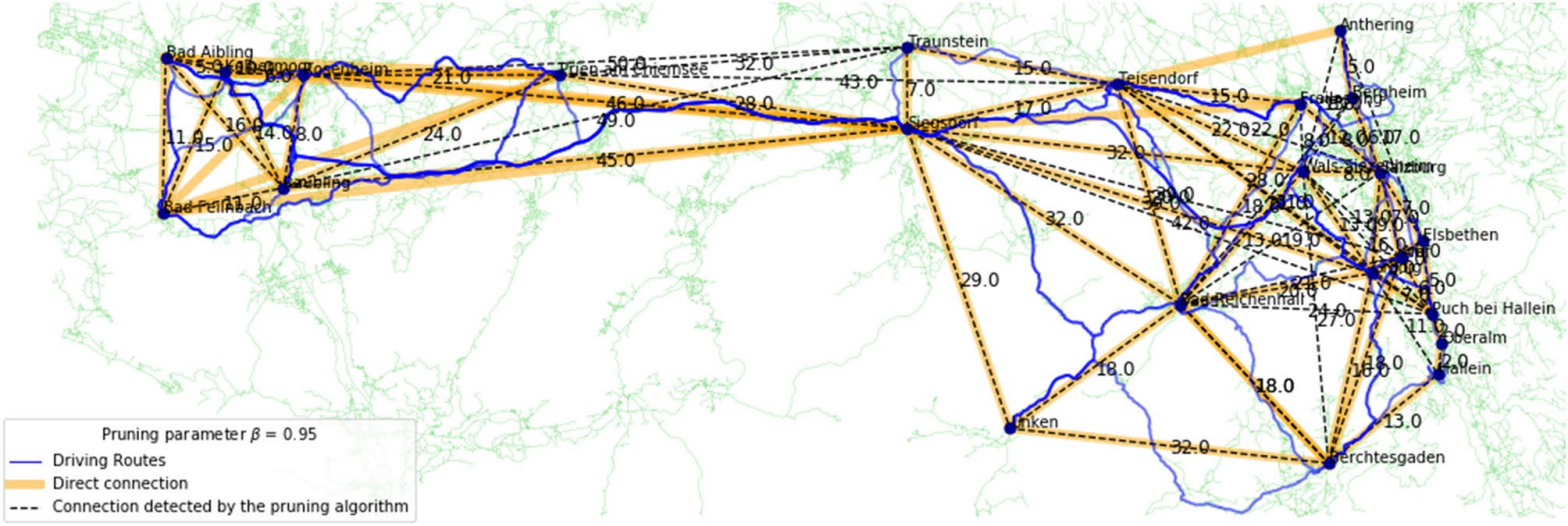
Connections of 23 locations in the border region between Germany and Austria with $\beta =0.95$.

**Figure 4:**
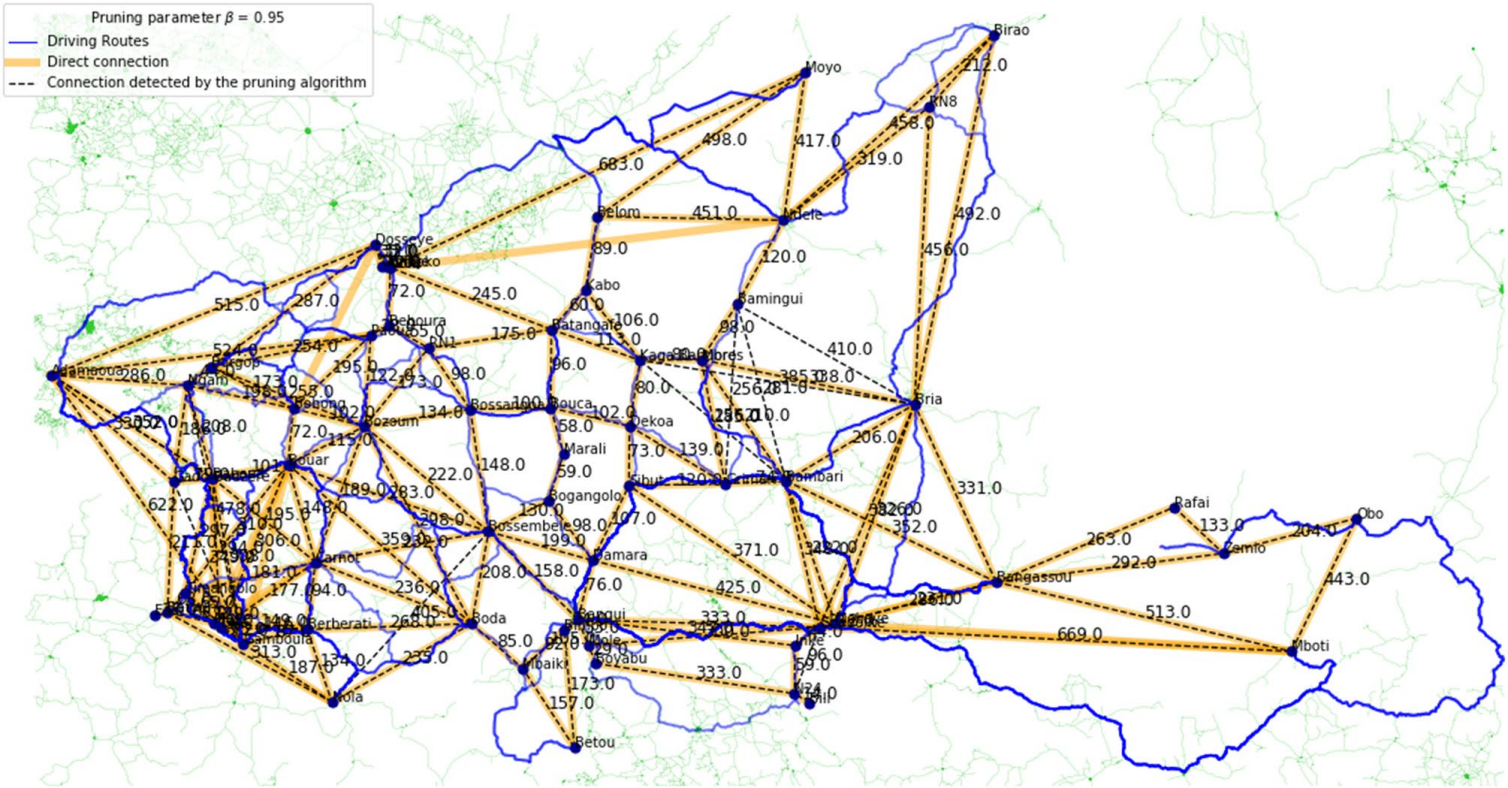
Connections of 62 locations in the Central African Republic with $\beta =0.95$.

In addition to computing quantitative performance measures, we visualised our results in Figs. 2,  3 and 4 (see also Supplementary Figure S1). These figures were generated using the OSMnx Python package, which is based on OSM to create, analyse, and visualise street networks^26^.

### Creation of the ground truth

We created the ground truth of direct driving connections for each of the four regions with OSM by inspecting if the fastest route (shortest time) between each location pair is direct. A connection between two locations is labelled direct if there is no other location on or nearby the fastest route. In most cases it was clear if a direct driving route between two locations exists, but there are also ambiguous situations (e.g., route from $l_1$ to $l_2$ is direct, but indirect from $l_2$ to $l_1$) and potential sources of error (e.g., small locations or refugee camps, especially in large regions, might not be marked explicitly in OSM), such that the creation of the ground truth was not straightforward (see Supplementary Note 1 for details). Even if the ground truth is created to the best of our knowledge, some uncertainty remains. Thus, the reported performance measures have to be interpreted accordingly.

### Federal state of Styria, Austria

For the region in the federal state of Styria in Austria, we extracted towns and cities within a rectangle with the geographic coordinates $N47.0 - N47.5$ and $E14.6 - E16.0$ from OSM. The OSM Overpass API^27^ returned 15 locations within this area, 14 towns and one bigger city, Graz. Therefore, the fully connected graph of this region contains 105 driving routes connecting the 15 locations. We obtained 29 direct driving routes between the 15 locations as the ground truth.

The fully connected graph was pruned with Algorithm 1, “Route Pruning for General Undirected Graphs” for several values of the pruning parameter $\beta$. Table 1 contains the results for the pruning parameters 0.9, 0.925, 0.95, and 0.975. For $\beta \in [0.9, 0.95]$, Precision, Recall and F1-Score are all above 0.9. Figure 2 visualises the results for $\beta =0.95$ with the established ground truth and pruned connections as well as the suggested fastest driving routes.

**Table 1:** Results for the triangle inequality pruned graph in Styria, Austria with 15 locations.

$\beta$	Ground truth routes	Routes after pruning	TP	FP	FN	Precision	Recall	F1-score
0.900	29	26	26	0	3	1.00	0.90	0.95
0.925	29	27	27	0	2	1.00	0.93	0.96
0.950	29	30	28	2	1	0.93	0.97	0.95
0.975	29	39	29	10	0	0.74	1.00	0.85

For $\beta = 0.95$, the pruning algorithm returns 30 direct driving routes between the 15 locations. 28 of the 29 ground truth routes are detected, it prunes one route that is part of the ground truth and declares two routes as direct connections that are not part of the ground truth. The route [Frohnleiten – Knittelfeld] in the central part of the region is 69 km long and is pruned from the fully connected graph, but is part of the ground truth. The algorithm detects the route [Frohnleiten - Leoben - Knittelfeld], which totals 72 km, and the route [Frohnleiten - Bruck an der Mur - Knittelfeld] which totals 71 km. As $72 \cdot 0.95 = 68.4 < 69$, the route [Frohnleiten - Knittelfeld] is pruned from the fully connected graph.

For this $\beta$, the algorithm also keeps two routes of the fully connected graph that are not part of the established ground truth. The first one is the route [Frohnleiten - Graz] in the southern part which passes by the location Gratwein-Straßengel on a highway, but not directly through the location. For this route, one could argue that it is direct because it does not go through the location, but we decided to not include it in the ground truth as the highway passes Gratwein-Straßengel very close by. The second route that the algorithm labels as direct, but that is not part of the ground truth, is the connection [Bruck an der Mur - Trofaiach] in the north. It is 26 km long and goes directly through Leoben, but not through the marked OSM position of Leoben. The distances of the respective single routes [Bruck an der Mur - Leoben] and [Leoben - Trofaiach] are 16 km and 12 km and add up to a total distance of 28 km. As $28 \cdot 0.95 = 26.6 > 26$, the algorithm declares the route [Bruck an der Mur - Trofaiach] as a direct one for $\beta = 0.95$.

### Border region between Germany and Austria

For the region around the German-Austrian border near Salzburg, we extracted towns and cities within a rectangular region that has the geographic coordinates $N47.6 - N47.9$ and $E12.0 - E13.1$ with the OSM Overpass API. This region has 23 locations, 22 towns and one bigger city, Salzburg. 12 locations are in Germany and 11 are in Austria. We computed the driving distance between each pair of locations with OSRM, which resulted in 253 driving routes, and established 57 direct routes connecting the 23 locations as the ground truth. The results for the pruning parameters between 0.90 and 0.95 are listed in Table 2 and the region is visualised for $\beta = 0.95$ in Fig. 3. The best F1-score is obtained with $\beta = 0.93$, while the best balance between Precision and Recall is obtained with $\beta =0.92$. In terms of the F1-score, the results for smaller and larger values of $\beta$ are still similar.

**Table 2:** Results for the triangle inequality pruned graph in the border region between Germany and Austria with 23 locations.

$\beta$	Ground truth routes	Routes after pruning	TP	FP	FN	Precision	Recall	F1-score
0.90	57	53	39	14	18	0.73	0.68	0.71
0.91	57	53	39	14	18	0.73	0.68	0.71
0.92	57	57	42	15	15	0.74	0.74	0.74
0.93	57	61	44	17	13	0.72	0.77	0.75
0.94	57	62	44	18	13	0.71	0.77	0.74
0.95	57	71	47	24	10	0.66	0.82	0.73

The area around the location Rosenheim in the western part of the region causes problems. The locations are connected via the fastest driving route (shortest time) and therefore they are often connected via the highway “Autobahn A8”. Using this road is the fastest connection between two locations in terms of time, but it is not the shortest route in terms of distance. For instance, the fastest route between the two locations Kolbermoor (west of Rosenheim) and Prien am Chiemsee (east of Rosenheim) is 33 km long and it takes 30 min via the Autobahn A8 according to OSM. An alternative route that takes more time uses the shortest distance between the two locations and passes directly through Rosenheim. The first intermediary route [Kolbermoor - Rosenheim] is a 6.1 km long country road that takes 11 min to drive. The second intermediary route [Rosenheim - Prien am Chiemsee] is a 21 km long country road that takes 22 min. Adding the two intermediary distances and driving times equals 27.1 km and 33 min, respectively, compared to the fastest driving route with 33 km and 30 min. The route [Kolbermoor - Prien am Chiemsee] will therefore always be removed from the fully connected graph by the route pruning algorithm, independent of the pruning parameter $\beta <1$, even though a direct, faster route exists.

### Central African Republic and neighbouring locations

As a third region, we chose a conflict scenario in the Central African Republic (CAR) which includes cities, towns and several refugee camps in CAR and in neighbouring countries. The 62 locations of this region are within the geographic coordinates $N2-N10.5$ and $E13-E27$, and the fully connected graph consists of 62 nodes and 1891 edges. For the ground truth, we detected 146 direct routes connecting the 62 locations. Table 3 summarises the results for the pruning parameters 0.80, 0.85, 0.90, 0.95, and 0.99. The pruning parameter $\beta = 0.95$ (see Fig. 4) returned the best result for this region with Precision, Recall and F1-score all above 0.9. After applying the route pruning algorithm on the fully connected graph, 149 routes are labelled as direct connections. 138 routes that are part of the ground truth are detected by the algorithm, 8 routes that are in the ground truth are not labelled as direct routes and 11 routes that are not part of the ground truth are labelled as direct routes by the algorithm.

**Table 3:** Results for the triangle inequality pruned graph for the Central African Republic with 62 locations.

$\beta$	Ground truth routes	Routes after pruning	TP	FP	FN	Precision	Recall	F1-score
0.80	146	104	104	0	42	1.00	0.71	0.83
0.85	146	116	115	1	31	0.99	0.79	0.88
0.90	146	126	124	2	22	0.98	0.85	0.91
0.95	146	149	138	11	8	0.93	0.95	0.94
0.99	146	193	146	47	0	0.76	1.00	0.86

In 3 of the 8 direct routes that were not detected by the algorithm, the location Mbile in the southwestern part of the region is involved, which is only 11 km away from the location Lolo. For instance, the route [Baboua - Mbile] is direct with a distance of 299 km. Adding up the distances of the routes [Baboua - Lolo] with 295 km and [Lolo - Mbile] with 11 km results in a total distance of 306 km. As $306 \cdot 0.95 = 290.7 < 299$, the route between Baboua and Mbile is pruned by the algorithm. For two other undetected direct routes, the distance is over 600 km. In the remaining three cases, direct connections between the two locations exist, but there are indirect routes that are only slightly longer.

For 5 of the 11 FPs, the routes go through the location Mbres, which is in the eastern part. The geographic coordinates of this location are off such that the five routes go through the location itself, but not the marked position in OSM. In the other 6 cases, the actual driving route is very close to other locations, such that they were not labelled as direct driving routes for the ground truth.

### South Sudan, Africa and locations in neighbouring countries

The fourth examined region is a conflict scenario in South Sudan, Africa, including several locations in neighbouring countries. The geographic coordinates of this region are approximately $N1 - N16$ and $E25 - E35$ and the fully connected graph has a total of 93 locations which are connected by 4278 edges. The ground truth of direct driving connections was created in two steps. In the first step, we obtained 142 direct routes connecting the 93 locations. There were several potential sources of error in the creation of the ground truth, especially for a region with many locations and several small refugee camps that are not marked explicitly in OSM. Thus, after considering the results of our automated location graph construction approach, this initial version of a ground truth was revisited. In this second pass, we discovered 178 direct routes between the locations and updated the ground truth by adding 46 direct routes and removing 10 routes that were found to be indirect.

In Table 4, we summarise the results for the pruning parameters 0.8, 0.85, 0.9, 0.95 and 0.99 with the updated ground truth. The pruning parameter $\beta = 0.95$ returned an F1-score over 0.9 with precision 0.86 and recall 0.95. After applying the route pruning algorithm on the fully connected graph, 197 routes were labelled as direct connections, of which 169 routes are also in the ground truth. 9 routes in the ground truth were missed by the pruning algorithm (see Supplementary Figure S1).

**Table 4:** Results for the triangle inequality pruned graph for the South Sudan case study with 93 locations.

$\beta$	Ground truth routes	Routes after pruning	TP	FP	FN	Precision	Recall	F1-score
0.80	178	134	134	0	44	1.00	0.75	0.86
0.85	178	149	146	3	32	0.98	0.82	0.89
0.90	178	162	156	6	22	0.96	0.88	0.92
0.95	178	197	169	28	9	0.86	0.95	0.90
0.99	178	326	175	151	3	0.53	0.98	0.69

For 9 of the 28 FPs, the route between the locations goes directly through another location in OSM. In most of these cases, the route does not go through the marked position of the intermediate location, but through the location itself such that these routes were labelled as indirect. The offset of the position marker adds enough distance to get a different result when applying Algorithm 1. For 17 connections, there is a third location nearby the route that is suggested by OSM such that they were not labelled as direct for the ground truth. The distance between locations is sometimes relatively big with more than 300 km. In such a case, if there was a location near the road (which, for these large distances can still be several kilometres), we declared this route as indirect. We might have been too conservative in the creation of the ground truth by labelling these routes as indirect. Thus, some of these 17 routes are worth discussing and could potentially also be part of the ground truth. For the remaining two connections, it was not perfectly clear if the routes are direct or indirect, as both involve a region where three refugee camps are within a small area (eastern part of the region). In both cases it was decided to label the routes as indirect, since they have a third location nearby the road that is taken, but one can also argue that they are actually direct.

Besides the 28 FPs, there are also 9 FNs. This could on the one side be due to some wrong entries in the ground truth (routes added that should not be in the ground truth) or due to the large distance between most of the locations pairs. For 7 instances, the distance between the locations is more than 700 km. In these cases another location could be relatively far off the route, but the pruning algorithm will eliminate it. One of these 7 routes is the connection [Rubkona - South_Darfur], which is 1434 km long in our records. It is therefore sufficient to find a third intermediary location that increases the total distance to less than 1509 km to not label it as a direct route with $\beta = 0.95$. Here, the location East_Darfur is causing the issue. The distance [Rubkona - East_Darfur] is 471 km and [East_Darfur - South_Darfur] is 954 km. Adding up those two gives a total distance of 1425 km, which is smaller than 1509 km such that the connection is removed. The remaining 2 routes were pruned because there is another location nearby the route.

### Runtime

To evaluate the performance of our approach, we benchmarked the multi-threaded C++ implementation of Algorithm 1 with naïve round-robin parallelization on a single Hewlett Packard Enterprise’s (HPE) Apollo node. The HPE Apollo system is equipped with two 64-core AMD EPYC 7742 CPUs and 256GB DRAM. The codes are available at https://github.com/djgroen/ExtractMap. In this benchmark, they were compiled with GCC 9.3 and linked against the latest version of OSRM C++ library, available from the master branch in the official GitHub repository of OSRM back-end. The distance matrix is calculated with the contraction hierarchies (CH) algorithm. The benchmark is performed on input OSM maps, downloaded from https://download.geofabrik.de. Locations correspond to the settlements from the OSM maps tagged with place equal to city, town, or village.

In Table 5, we summarise results of the benchmark on the level of countries and continents. Despite cubic complexity, Algorithm 1 performs well on the real world applications. We also demonstrate that our implementation of Algorithm 1 in Table 5 allows to construct location graphs for $\sim$10k locations on the route networks of the entire continents in reasonable time. In all benchmarks, the multi-core implementation of the pruning step takes order of magnitude less time than the construction of the distance matrix where we used highly optimized multi-threaded OSRM library.

**Table 5:** Performance of the triangular pruning for the undirected routing table.

Region	Number of locations	Routing	Pruning	
			Serial	128 cores
South Sudan (all settlements)	1783	3.15 s	1.74 s	43.38 ms
Africa (cities/towns)	9807	130.01 s	585.89 s	62.22 s
Australia & Oceania (cities/towns)	1693	119.38 s	–	49.78 ms
Europe (cities/towns)	18,091	5.01 h	–	316.36 s
North America (cities/towns)	9959	3.06 h	–	72.78 s
South America (cities/towns)	8591	29.38 m	–	71.72 s
Central America (cities/towns)	1948	66.303 s	–	41.12 ms

Note that, similar to Floyd-Warshall all-pairs shortest path algorithm^28^, Algorithm 1 enables applying cache-oblivious^29^ and communication-avoiding^30^ speed-up techniques to give better cache locality and reduce communication complexity of the basic algorithm. Moreover, since in contrast to Floyd-Warshall, Algorithm 1 is embarrassingly parallel in terms of triangle traversal, it has higher potential for improving cache locality and reducing communication costs.

## Discussion and limitations

In this work, we produce optimal location graphs by proposing a computationally efficient two-step approach: in the first step, pairwise distances between locations of interest are computed with state-of-the-art batched shortest path algorithms, such as MLD or CH in a time complexity of $\mathscr {O}((|E_G|+L_G \log L_G ) L)$. In the second step, these pairwise paths are then pruned with Algorithm 1 in a time complexity of $\mathscr {O}(L^3)$.

Introducing the parameter $\beta$ to Algorithm 1 further adds flexibility to our approach, making it applicable to both lossy edge pruning ($0<\beta <1$) in the spirit of Zhou et al.^9,10^ or the creation of location graphs with additional indirect routes ($\beta >1$). As our results show, the location graphs constructed using our two-step approach agree well with manually created location graphs. In three of the four case studies we achieved F1-scores exceeding 0.9, and the runtime of the pruning algorithm is still acceptable even for thousands of locations, for which a manual creation of the location graph would be infeasible.

We have made the general observation that small values of $\beta$ lead to strong pruning, i.e., large Precision and, if direct routes are removed, small Recall. In contrast, large values of $\beta$ imply conservative pruning, resulting in large Recall and, if too many indirect routes are kept, small Precision (this will continue to hold naturally if $\beta$ exceeds 1). While we have observed that the highest F1-scores are achieved for $\beta \in [0.9, 0.95]$ in all four scenarios, the optimal value depends not only on the geographical region (and the degree to which a road network is established), but also on the type of locations (major cities vs. small villages). This dependence on the general road infrastructure is also reflected in the runtime experiments (in Table 5), which show vastly different routing times for Africa, South America, and North America despite similar numbers of locations.

We have observed that, even with careful tuning of $\beta$, the resulting location graph may still differ from a manually created ground truth. Especially for routes with a long distance between a location pair, the multiplicative factor $\beta$ may result in pruned direct routes if a third location is close to this direct route. We have seen such examples in the CAR and the South Sudan case studies. We believe that similar considerations will hold for routes with short distances if the multiplicative factor is replaced by an additive factor, as suggested at the end of the Methods section. Therefore, the selection of these hyperparameters always has to be guided by the application setup (structure of the road network and distribution of locations), application requirements (sparse and lossy or dense and redundant location graphs), and by results from cross-validation.

However, we believe that such inaccuracies do not appear as roadblocks in many of the applications for which location graphs are required. Considering the example of forced migration simulation with agent-based models from Suleimenova et al.^8^, the existence of indirect routes in $\mathscr {G}$ is less problematic than missing routes, ensuring that the location graph is connected. Moreover, considering the multi-graph nature of the actual road network and the fact that the algorithm may prune direct routes when locations are close to each other or close to a direct connection, we argue that these errors are acceptable as long as the path distance between a set of locations in $\mathscr {G}$ is within a reasonable range to the actual road distance between these locations, cf. Eq. (1). Since some of the mentioned limitations are also shared by other graph pruning algorithms^9,10,25^, we are convinced that the improved computational complexity, the added flexibility due to the hyperparameter $\beta$, and the remarkable performance of our approach as confirmed in our experimental study present a valid contribution.

## Supplementary Information

Supplementary Information.
