# Supplementary material for: A route pruning algorithm for an automated geographic location graph construction
Source: Sci Rep. 2021 Jun 2;11:11547. doi: 10.1038/s41598-021-90943-8 (PMC8172915; doi:10.1038/s41598-021-90943-8)
Supplement: Supplementary file 1 — Supplementary Information. [file 41598_2021_90943_MOESM1_ESM.docx]

**Supplementary Information for “A route pruning algorithm for an automated geographic location graph construction”.**

**Christoph Schweimer**^1^**, Bernhard C. Geiger**^1,*^**, Meizhu Wang**^1^**, Sergiy Gogolenko**^2^**, Imran Mahmood**^3^**, Alireza Jahani**^3^**, Diana Suleimenova**^3,**^**, and Derek Groen**^3,4^

^1^Know-Center GmbH, Graz, Austria
^2^High Performance Computing Center, Stuttgart, Germany
^3^Department of Computer Science, Brunel University London, London, UK

^4^Centre for Computational Science, University College London, London, UK

^*^ geiger@ieee.org
^**^ diana.suleimenova@brunel.ac.uk

This document serves as Supplementary Information for the paper “A route pruning algorithm for an automated geographic location graph construction”.

**Supplementary Note 1. Creation of the Ground Truth**

We briefly describe some of the situations, which do not have straightforward ground truth

creation:

1. Bigger locations are critical. A route between a pair of locations can go through another location within its city boundaries, but not through the marked position in OSM, which is often the city centre. In these cases it was decided to label the route as indirect, as it technically goes through another place.

2. Certain locations are close to a main road, but the route between two other locations does not pass directly through the location. If the route between two other locations uses that road, it was decided to declare the route as indirect, as the distance to the in-between location is negligible and the location is en-route.

3. For some connections, it makes a difference if a route is taken from location A to B or the other way round. In the Styria example, the route [Frohnleiten - Knittelfeld] is direct and we included it in the ground truth. For the connection [Knittelfeld - Frohnleiten] a different route is suggested which goes through two other locations, Leoben and Bruck an der Mur, and should therefore be labelled indirect. In these cases, we decided to include the route between a location pair in the ground truth because a direct fastest connection exists.

4. Regions where many locations are within a small area also led to ambiguities. In the eastern part of the South Sudan example, there is the location Jewi in Ethiopia and roughly 50 km to the West of this location, there are the three locations Kule, Nguenyyiel and Tierkidi within a small radius. For connections that include one of these locations, it was not perfectly clear which ones are direct.

5. In several cases, small locations or refugee camps lie on the route between two locations, but they are often not marked explicitly as locations in OSM. It might have happened that routes were labelled as direct routes, although they are passing through or close by one of these small places.

6. In the South Sudan case study, there are more than 90 locations, such that we could have potentially overlooked a location that lies between two other locations and label a route as direct.

7. In certain regions, the road network is underdeveloped. Two locations might be close to each other on the map, but no short direct connection between them exists or there is no road that goes to the marked position of a location in OSM.

**Supplementary Note 2. Overview of the South Sudan network map**


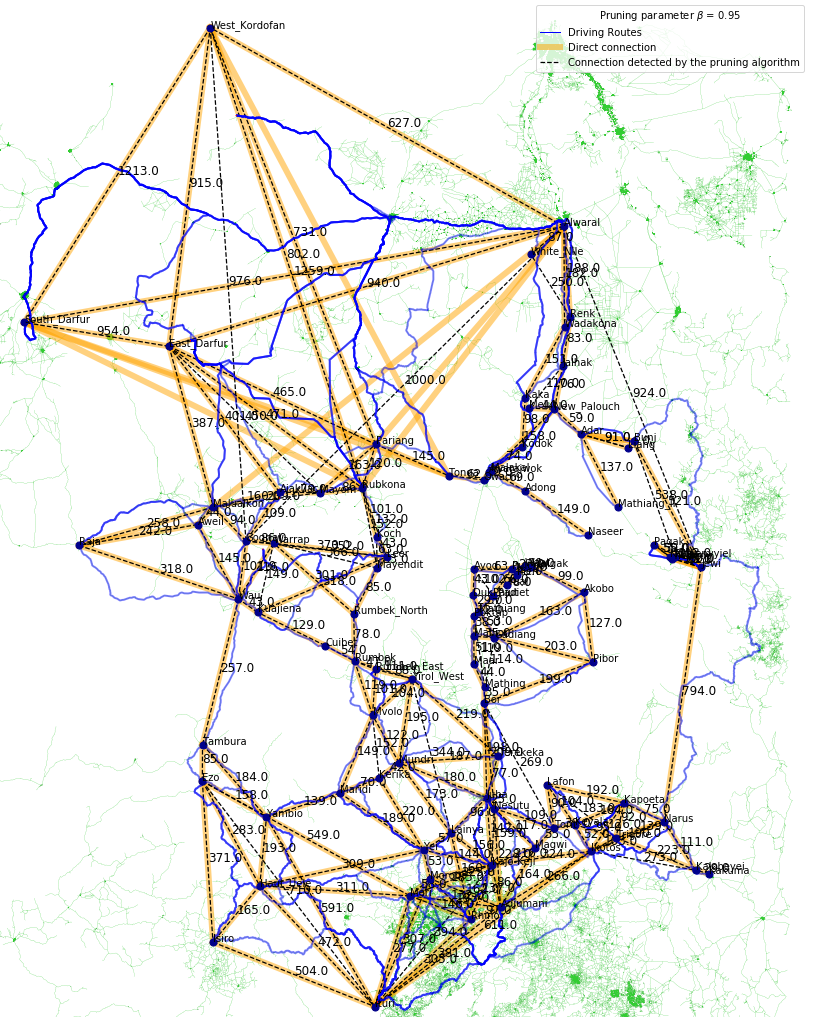


Figure S1. Connections of 93 locations in South Sudan with β = 0.95
